# Comparative genomics analysis of pKF3-94 in *Klebsiella pneumoniae* reveals plasmid compatibility and horizontal gene transfer

**DOI:** 10.3389/fmicb.2015.00831

**Published:** 2015-08-18

**Authors:** Jianchao Ying, Songquan Wu, Kaibo Zhang, Ziqiang Wang, Wen Zhu, Mei Zhu, Ying Zhang, Cong Cheng, Huifeng Wang, Huifen Tou, Chuanxin Zhu, Peizhen Li, Jun Ying, Teng Xu, Huiguang Yi, Jinsong Li, Liyan Ni, Zuyuan Xu, Qiyu Bao, Junwan Lu

**Affiliations:** ^1^Institute of Biomedical Informatics/Zhejiang Provincial Key Laboratory of Medical Genetics, Wenzhou Medical UniversityWenzhou, China; ^2^School of Medicine, Lishui CollegeLishui, China; ^3^National Institute of Biological SciencesBeijing, China; ^4^Wenzhou Center for Disease Control and PreventionWenzhou, China; ^5^The Second Affiliated Hospital, Wenzhou Medical UniversityWenzhou, China

**Keywords:** plasmid compatibility, comparative genomics, horizontal gene transfer, *Klebsiella pneumoniae*, plasmid

## Abstract

In order to get insights into plasmid evolution and the dissemination of multidrug resistance, we performed extensive comparative genomics analyses of the *Klebsiella pneumoniae* plasmid pKF3-94 and some of its related plasmids. pKF3-94 is one of three plasmids isolated from the *K. pneumoniae* strain KF3. Of the 144 putative genes it harbors, 69 can be functionally assigned to be involved in transfer conjugation, transfer leading, antimicrobial resistance, transposon function, and plasmid replication. Comparison of plasmid replicon sequence types revealed that pKF3-94 carries two replicons that are distinct from those carried on the two sibling *K. pneumonia* plasmids pKF3-70 and pKF3-140, thereby allowing pKF3-94 to coexist with these latter plasmids in the same host cell. Comparative genomics analyses further showed that pKF3-94 is more similar to plasmids pK1HV and pC15-k, which were isolated from different *K. pneumonia* strains, than to pKF3-70 and pKF3-140. Interestingly, pK1HV contains a unique 49 kb region rich in mobile genetic elements and drug resistance genes, while pKF3-94 and pC15-k share a 15 kb homology region partitioned into a region rich in drug resistance genes and one containing a replicon. It is conceivable, therefore, that pK1HV and pC15-k have both arisen from a common pKF3-94-like plasmid. The comparisons lend further support for the role horizontal gene transfer plays in genome evolution and in the dissemination of genetic elements including drug resistance genes.

## Introduction

Plasmids are versatile genetic elements that participate in various processes including the shuttling of genetic information between different hosts. This process, called horizontal gene transfer (HGT), can rapidly introduce newly evolved donor genes into a variety of host genomes (Jain et al., 2003). HGT mechanisms include transformation, conjugation, transduction, and variations of these as well as the role of mobile genetic elements (MGE) (Brigulla and Wackernagel, 2010). Conjugation is driven by MGE which can be conjugative plasmids or conjugative transposons (Brigulla and Wackernagel, 2010). MGEs generally carry determinants for their translocation and maintenance (Bahl et al., 2009) and are often beneficial to the host cell by protecting, for instance, against antibiotics, heavy metals, or extreme environmental conditions (Sobecky and Coombs, 2009). In fact, by their capacity to transfer diverse genetic information between bacteria, conjugative plasmids critically contribute to bacterial genome evolution (Périchon et al., 2008).

A limitation to HGT, however, is the phenomenon of plasmid incompatibility. Plasmid incompatibility is generally defined as the failure of two co-resident plasmids to be stably inherited in a single host cell in the absence of external selection (Novick, 1987). It is due to sharing of one or more elements of the plasmid replication or partitioning systems rather than to any specific incompatibility (inc) gene (Novick, 1987). By definition, plasmids incompatible with each other belong to the same incompatibility group (Couturier et al., 1988). In multi-replicon plasmids, potential incompatibility determinants depend on combinations of distinct replicons. Multi-replicon plasmids can allow a host cell to acquire plasmids carrying incompatible replicons when replication is driven by compatible replicons (Villa et al., 2010). The classic multi-replicon IncF plasmid contains the FII replicon regulated by CopA, a constitutively synthesized 90 nt antisense-RNA, which is normally silent, and FIA and FIB replicons, which function only in enteric bacteria and are regulated by iterons, in cis-negative binding sites of the replication protein RepA. In plasmids F and p307, the FII replicon is substituted by a non-functional FIC replicon, while plasmids R1 and R100 of *E. coli* contain only one functional FII replicon (Villa et al., 2010). FII replicons are more divergent than FIA, FIB, and FIC replicons, and consequently can be divided into FII, FII_K_ (identified in K*. pneumoniae*), FII_S_ (identified in *Salmonella spp*.), FII_Y_ (identified in *Y. pestis, Y. pseudotuberculosis*, and *Yersinia enterocolitica*), FII_SH_ (identified in *Shigella flexneri* and *Shigella sonnei*). It has been demonstrated that, for instance, FII replicons do not participate in the initiation of replication of a plasmid when they are associated with FIA and/or FIB replicons, and hence, the presence of these latter replicons can overcome the incompatibility barrier between IncF plasmids (Villa et al., 2010). Plasmids of the IncF group represent one of the most frequent plasmid types and they often carry more than one replicon promoting the initiation of replication (Villa et al., 2010; Dolejska et al., 2013).

Plasmid-mediated transfer of drug-resistance genes is considered one of the most important mechanisms for the spread of multidrug resistance of pathogens (Zhao et al., 2010; Bai et al., 2013). For instance, from the nosocomial pathogen *Klebsiella pneumoniae*, which causes pneumonia and urinary tract infections particularly in patients in intensive care units (Landman et al., 2007), more than 100 plasmids ranging from 1.3 to 317 kb have been isolated and sequenced. Some of them were isolated from the same strain, such as pKDO1 (127 kb) and pKPN-CZ (207 kb) from *K. pneumoniae* ST416 (Dolejska et al., 2013); or pPKPN1 (283 kb), pPKPN2 (103 kb), pPKPN3 (70 kb), and pPKPN4 (6 kb) from *K. pneumoniae* PittNDM01 (Doi et al., 2014). The conjugative plasmid pKF3-94 of the IncF group was isolated from *K. pneumoniae* strain KF3, which contained in addition pKF3-70 and pKF3-140 (Zhao et al., 2010). Plasmid pKF3-94 exhibits a low sequence identity with either pKF3-70 or pKF3-140 but is highly similar to pK1HV from *K. pneumoniae* strain K1HV and to pC15-k from *K. pneumoniae* strain 997. pKF3-94 not only encodes the antimicrobial resistance genes *bla*_CTX−M−15_ and *bla*_TEM_, but also carries two replicons. Here, we analyzed the replicon sequence types of these plasmids (Plasmid RST) to explain the mechanism of plasmid compatibility by using the Plasmid MLST Database (http://pubmlst.org/plasmid/). Identifying Plasmid RST contributes to recognizing and sub-categorizing plasmids, and further to analyzing the distribution of plasmid replicon and discovering their evolutionary origin (Villa et al., 2010). Moreover, we determined the genomic structure of pKF3-94 and performed evolutionary and extensively comparative analysis among the plasmid pKF3-94 and the related plasmids in order to evaluate the origin and evolution of these plasmids from *Klebsiella pneumoniae* and the distribution of multidrug resistance genes.

## Results and discussion

### General features of pKF3-94

**Figure 1:**
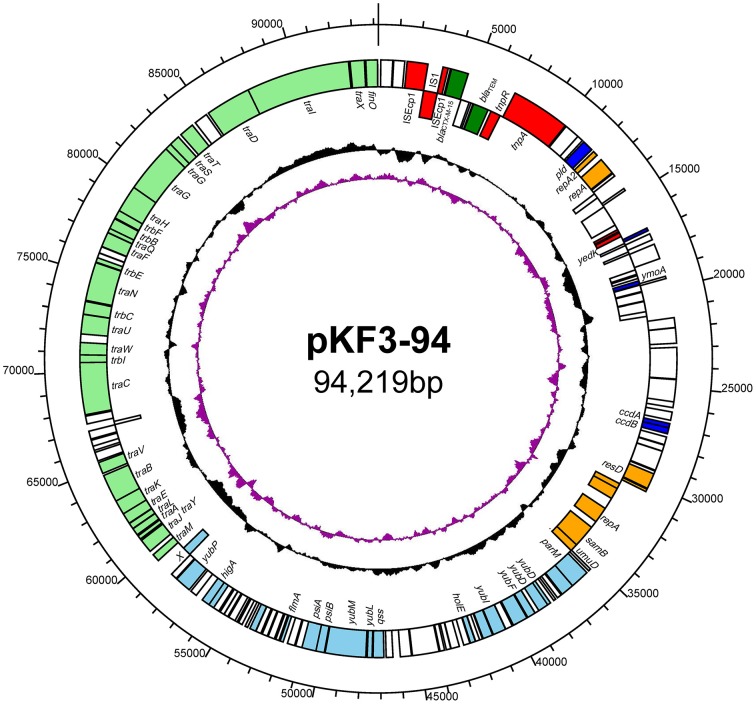
**The circular map of pKF3-94 genome**. Counting from outside toward the center, the first circle refers to the position in bp. The second circle marks genes encoded on the leading strands (outwards) or lagging strands (inwards). The different functional regions are shown in different colors; green, drug-resistance related genes; red, transposase/insertion sequences; orange, replicon related genes; light blue, transfer leading regions; light green, transfer conjugation regions; blue, genes with other functions; blank, genes with unknown functions. The third circle shows GC content with an average of 50%, whereby a G+C content of more than 50% is shown toward the outside and a G+C content of less than 50% toward the inside. The fourth circle shows GC skew (G–C/G+C) with a positive GC skew toward the outside and a negative GC skew toward the inside.

Plasmid pKF3-94 is a circular 94,219 bp plasmid with an average GC content of 51.6% (Figure 1). We identified 144 putative open reading frames (ORFs), with the majority of them (119/144) transcribed from the leading strand. Sixty nine ORFs were predicted to encode proteins with known functions, including transfer conjugation, transfer leading, antimicrobial resistance, transposase function, replicon functions, and other functions (Figure 1).

### Replicon sequence types of pKF3-94, pKF3-70, and pKF3-140

pKF3-94 and pKF3-140 are both multi-replicon plasmids, while pKF3-70 only carries one replicon. As shown in Table 1, pKF3-70 belongs to the incompatibility group FII, as it carries the FII_2_ replicon allele (F2:A-:B-). pKF3-140 carries three different replicons, FII_1_, FIA_2_, and FIB_2_ (F1:A2:B2); pKF3-94 carries two replicons; although the Plasmid RST program identified only one FII_K2_ replicon allele, (K2:A-:B-). The replication initiation protein (RepA) of FII_K2_ shares 83% sequence similarity with that of FII_2_/FII_1_. The amino acid sequence of the RepA of FII_2_, however, shows 98% similarity with that of FII_1_. The unassigned replicon (unknown replicon type and not belonging to IncF family) of pKF3-94 is only found in 11 other plasmids, including pK1HV, pNJST258C3, pNJST258N3, and CCBH10892 (an unamed plasmid from *E. hormaechei*, Table 1). The corresponding *repA* genes in these plasmids are >99% identical with each other. It is noteworthy that pNJST258C3 and pNJST258N3 are single-replicon plasmids, and so it is likely that this unassigned replicon has the function of replication initiation.

**Table 1:** **Plasmids/strains and their replicons identified in this study**.

**Plasmid/strain**	**Bacterium**	**Allele numbers of the replicons**			**FAB formula**
		**FII,FII_*K*_,FII_*S*_**	**FIA**	**FIB**	
pKF3-70	*K. pneumoniae*	F2	–	–	F2:A-:B-
pKF3-94	*K. pneumoniae*	K2	–	–	K2:A-:B-
pKF3-140	*K. pneumoniae*	F1	A2	B2	F1:A2.B2
pK1HV	*K. pneumoniae*	K2	–	–	K2:A-:B-
pC15-k	*K. pneumoniae*	K2	–	–	K2:A-:B-
pNJST258C3	*K. pneumoniae*	–	–	–	–
pNJST258N3	*K. pneumoniae*	–	–	–	–
CCBH 10892 plasmid	*E. hormaechei*	–	–	–	–
pKP1780–kpc	*K. pneumoniae*	K2	–	–	K2:A-:B-
SA20094177	*S. enterica*	S1	–	B22	S1:A-:B22

Although pKF3-94, pKF3-70, and pKF3-140 each carry one replicon of the incompatibility group FII, they belong to different FII replicon alleles and possess different origins. In particular, FII_K2_ is different from the other two FII replicons (Villa et al., 2010). FII_K2_ is widely distributed in the plasmids from *K. pneumoniae*, while FII_1_ and FII_2_ are derived from *E. coli*. In addition, because FIA_2_ and FIB_2_ are also present in pKF3-140, FII_1_ does not participate in the initiation of replication in this plasmid (Villa et al., 2010). Therefore, these three plasmids could coexist in the same bacterial cell, avoiding the potential incompatibility resulting from carrying similar replicons.

### Comparative genomics analysis of pKF3-94 and other plasmids

**Figure 2:**
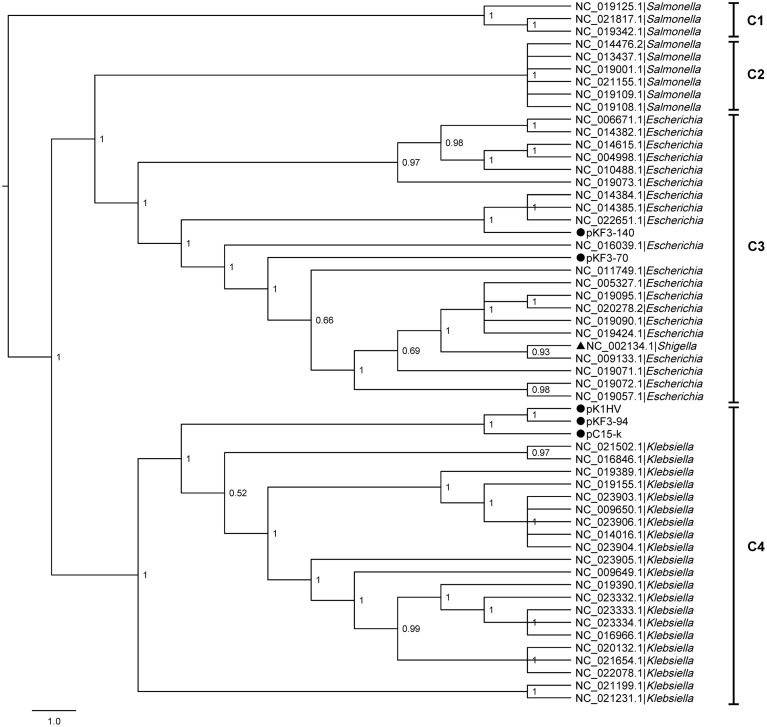
**The phylogenetic tree of 56 plasmids including pKF3-94, pKF3-70 and pKF3-140**. The tree can be divided into four clades: C1, C2, C3, and C4. Every clade was supported by Bayesian posterior probabilities (BPP).

To explore the phylogenetic relationship of pKF3-94 to plasmids from different bacteria, we performed a comparative genomics analysis of the corresponding plasmid sequences available in the NCBI database. A phylogenetic tree was constructed based on the six homologous genes concatenated sequences of 56 plasmids including pKF3-94, pKF3-70, and pKF3-140 using the six homologous genes concatenated sequences (Gadagkar et al., 2005, Figure 2). The phylogenetic relationship shows that plasmids from the hosts of the same genus cluster together. For instance, the plasmids pKF3-94 clusters together with other *Klebsiella* plasmids (Figure 2, C4), but pKF3-70 (Yi et al., 2010) and pKF3-140 (Bai et al., 2013) cluster together with plasmids from *Escherichia* (Figure 2, C3), but not with other plasmids from *Klebsiella* such as pKF3-94. This suggests that the origin of pKF3-94 is different from that of either pKF3-70 or pKF3-140. In addition, we found that pKF3-94 clusters closer together with pC15-k and is located on the same branch with pK1HV (Figure 2, C4). A collinearity analysis of the plasmid genomes including pKF3-94, pK1HV, pC15-k, pKF3-70, and pKF3-140 was also performed (Supplementary Figure S1). This suggests that pKF3-94 might have a closer phylogenetic relationship with pK1HV and pC15-k than with pKF3-70 and pKF3-140.

**Figure 3:**
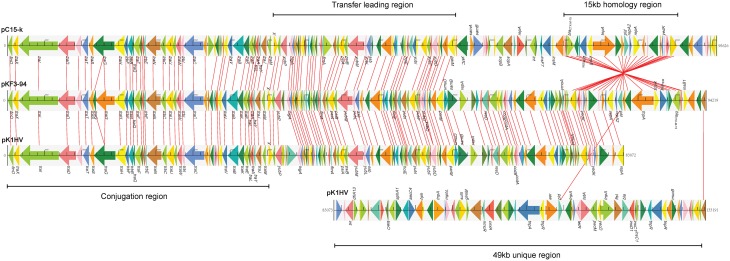
**Comparison of genome structure of the plasmids pKF3-94, pK1HV, and pC15-k**. Homologous genes are marked with the same color and connected by lines, while non-homologous genes are left unconnected.

Further comparative genomics analysis showed that pKF3-94 is about 39 kb smaller than pK1HV (133 kb), but of similar size compared to pC15-k (95 kb). Nevertheless, 83.3% (120/144) and 68.8% (99/144) of the genes of pKF3-94 have a high degree of similarity (>90%) with those of pK1HV and pC15-k, respectively. Furthermore, the three plasmids share a conserved backbone sequence, which includes transfer conjugation and transfer leading regions, and they possess their own variable regions, which mainly include antimicrobial resistance genes and insertion sequences (Figure 3).

### Replicon regions of pKF3-94 and pK1HV and the 49 kb unique region of pK1HV

**Figure 4:**
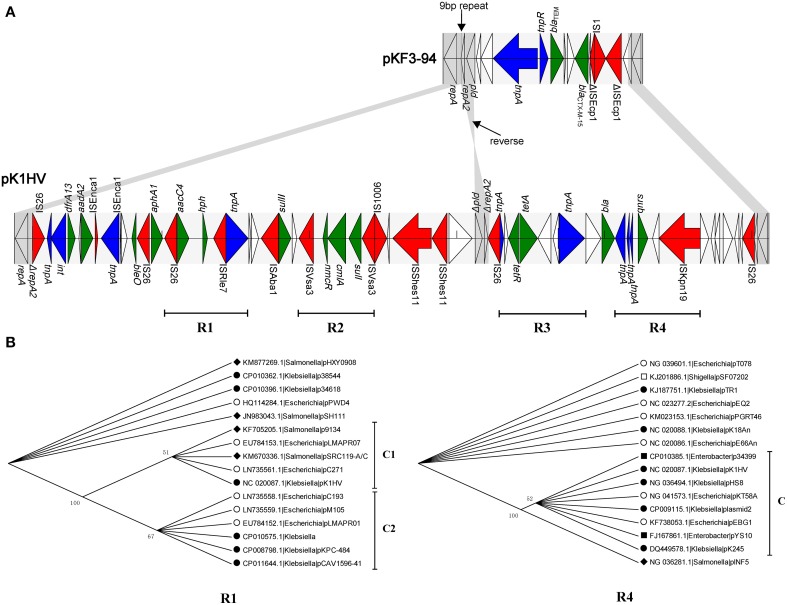
**Comparison of the structure of the 49 k unique region of pK1HV with the corresponding region of pKF3-94**. **(A)** The identical sequence regions are connected with gray bars. The different functional elements are labeled in different colors, with insertion sequences in red, transposase genes in blue, drug resistance genes in green, and the remaining genes left blank. The phylogenetic trees of R1 and R4 **(B)**.

The three plasmids pKF3-94, pK1HV, and pC15-k each carry two replicons, with FII_K2_ co-existing in all of them. The unassigned replicon of pKF3-94 is also present in pK1HV, but not in pC15-k. The FII_K2_ replicon consists of *repA* (858 bp, encoding RepA) and *repA2* (252 bp, encoding the replication regulatory protein RepA2) in pKF3-94, while the FII_K2_ replicon in pK1HV is incomplete and includes *repA* and only 17 bp of a truncated *repA2* gene. Moreover, the *pld* gene (561 bp, encoding phospholipase D) downstream of *repA2* of pKF3-94 also exists in pK1HV, but is incomplete (480 bp, designated Δ*pld*) and in a reverse orientation (Figure 4A). Comparative analysis of the flanking sequences of Δ*pld* indicates that the sequence downstream of Δ*pld* of pK1HV includes a truncated *repA2* gene (244 bp), with an intervening sequence identical to that of pKF3-94 (a total of 379 bp in length). In addition, there are 9 bp perfect direct repeats (DRs), each lying downstream of the two parts of Δ*repA2* in pK1HV (Figure 4A). This arrangement suggests that recombinations have occurred between the inverted Δ*pld* and Δ*repA2* sequence in pK1HV. The region downstream of *pld* of pKF3-94 seems to be a hot spot of recombination. The resistance genes (*bla*_CTX−M−15_ and *bla*_TEM_) and two transposase genes (*tnpA* and *tnpR*) are clustered in this region. The sequences flanking Δ*pld* in pK1HV are about 49 kb long and are also littered with MGEs and antimicrobial resistance genes.

Further, we identified 15 complete or incomplete insertion sequences, 9 transposase/integrase genes and 14 antimicrobial resistance genes in this region. What's more, we found four relatively complete underlying structure of transposons (Figure 4A, R1, R2, R3, and R4). They have similar characteristics that the resistant genes are flanked by several insertion sequences/transposases. In addition, a phylogenetic analysis was also performed for these regions. For example, *aacC4* (coding aminoglycoside N(3′)-acetyltransferase IV) and *hph* (coding hygromycin-B phosphotransferase) are flanked by insertion sequences IS26 and ISRle7 in R1. This region is only present in 16 plasmids covering 3 genera (*Escherichia, Klebsiella* and *Salmonella*). The phylogenetic analysis showed that the branch of pK1HV and the other 4 plasmids (Figure 4B, C1) cluster together with that of other 6 plasmids (Figure 4B, C2). It suggests that they probably have the same origin. While in R4, *qnrS* (coding fluoroquinolone resistance protein) is surrounded by ISKpn19 and *tnpA* (IS2 transposase). *qnrS* along with its adjacent insertion sequences are present in 16 plasmids covering 5 genera (*Escherichia, Klebsiella, Enterobacter, Salmonella*, and *Shigella*). R4 of pK1HV clusters together with other 7 plasmids (Figure 4B, C), and they also have a closer phylogenetic relationship with the plasmid pINF5 from *Salmonella* than with other 7 plasmids. It is not difficult to see that these transposons (R1, R4 and including R2 and R3 in Supplementary Figure S2) are uniformly distributed among these genera (*Escherichia, Klebsiella*, and so on). Furthmore, it suggests that horizontal gene transfer of these transposons probably happens widely among strains, species or genera, even some phylogeneticly remote bacteria. This finding suppports the notion that this region corresponds to a hot spot of recombination, whereby the antimicrobial resistance genes have likely been inserted with the help of MGEs.

### Comparative genomics analysis of transfer leading and transfer conjugation regions between pKF3-94 and the related plasmids

**Figure 5:**
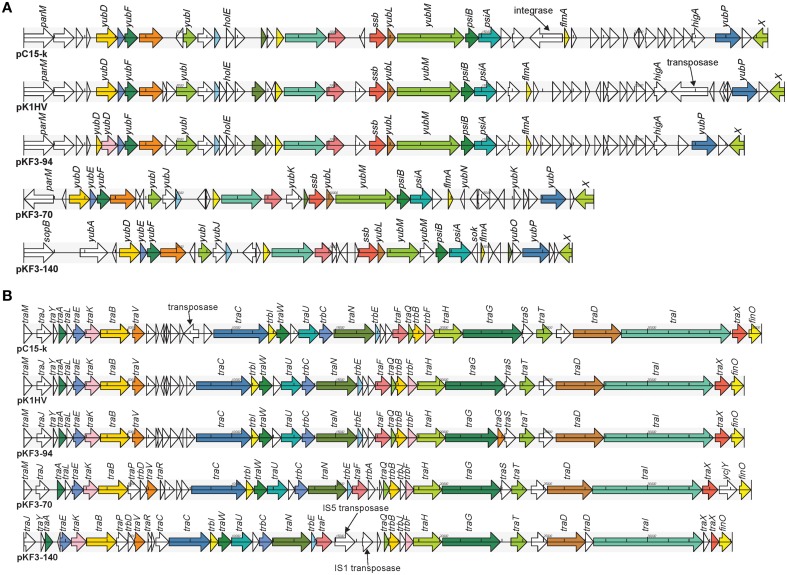
**Comparison of genome structure of the transfer leading (A) and conjugation regions (B) of pKF3-94, pK1HV, pC15-k, pKF3-70, and pKF3-140**. The homologous genes present in the five plasmids are marked with the same colors, while the non-homologous genes are left blank.

The 23.6 kb transfer leading region of pKF3-94 is the fragment of DNA firstly transferred into the recipient bacterium during the conjugation process (Manwaring et al., 1999). It contains a large number of ORFs with unknown function and genes involved in plasmid stability, including *ssb* (encoding plasmid-derived single-stranded DNA-binding protein), *flmA* (encoding stable plasmid inheritance protein), and *psiA* and *psiB* (genes related to plasmid SOS inhibition) (Figure 5A). Of the 48 ORFs in the transfer leading region of pKF3-94, 47 were found to be similar to those of pK1HV (the similarity of amino acid sequences are between 92.11 and 100%, except for two ORFs where they are 49.23 and 74.14%, respectively). Fourty-two of the 48 ORFs are similar to those of pC15-k (the similarity of amino acid sequences are between 88.46 and 100%). In addition, compared with the sequence of pKF3-94, there are some insertions in pK1HV and pC15-k, such as the insertion sequence (including a transposase gene) downstream of *higA* of pK1HV and the insertion sequence (including an integrase gene) upstream of *flmA* of pC15-k. Despite these insertions, the backbone of this region (including 14 genes) remains unchanged. This suggests that the transfer leading regions of pKF3-94, pK1HV, and pC15-k share a common origin. Nevertheless, the transfer leading region of pKF3-94 is more dissimilar to those of pKF3-70/pKF3-140 compared with to those of pC15-k/pK1HV. Of the 48 ORFs encoded on the transfer leading region of pKF3-94, only 21 correspond to homologous genes in pKF3-70 (the similarity of amino acid sequences is between 36.29 and 80.26%), and only 19 correspond to homologous genes in pKF3-140 (the similarity of amino acid sequences is between 36.40 and 86.84%). This further supports the hypothesis that pKF3-94 share a closer phylogenetic relationship with pK1HV and pC15-k than with the plasmids of pKF3-70 or pKF3-140. Moreover, these five plasmids share 18 homologous genes in the transfer leading regions (Figure 5A).

In pKF3-94, the 34.6 kb conjugation region lies adjacent to the transfer leading region and encodes 40 CDSs. This region harbors 22 *tra* genes (*traM, traJ* etc.), 5 *trb* genes (*trbI, trbC* etc.), and a conjugal transfer repressor gene, *finO* (Figure 5B). The conjugation region of pK1HV is almost identical with that of pKF3-94, with a nucleotide sequence identity of 99.89% and sharing 38 homologous genes (the similarity of amino acid sequences were all above 99.57%). pC15-k shows a nucleotide sequence similarity of greater than 99% with pKF3-94 and shares 38 homologous genes (amino acid sequence similarities are all above 99.88%), although an insertion (a transposase gene with a size of 826 bp) upstream of *traC* was identified in pC15-k compared with the corresponding regions of pK1HV and pKF3-94. This suggests that the conjugation region of these three plasmids might have a common origin. Unexpectedly, the genome structure of the conjugation regions of pKF3-70 and pKF3-140 is also similar to that of pKF3-94. However, the degree of similarity of the homologous genes in this region of pKF3-94 and pKF3-70 or pKF3-140 is far lower than that of the corresponding regions of pKF3-94 and pK1HV or pC15-k. Twenty-nine of the 40 genes in pKF3-94 share an amino acid sequence similarity above 22.75% with pKF3-70, and only 16 genes show a similarity of 60% or more (with 85.19%, *traT* has the highest similarity). Between pKF3-94 and pKF3-140, there are 26 genes with similarity above 31.15%, and only 14 genes share a similarity above 60% (with 84.21%, *traT* has again the highest similarity). There are 23 homologous genes in these five plasmids (Figure 5B). In addition, we found that the conjugation region of pKF3-70 and pKF3-140 are more similar to each other than to that of pKF3-94. For example, we found an insertion of *traP* and *trbD* between *traB* and *traV* in both pKF3-70 and pKF3-140 and an insertion of *trbA* or IS5-transposase and IS1-transposase between *traF* and *traO* in pKF3-70 or pKF3-140, respectively. Nevertheless, pKF3-70 and pKF3-140 also have their distinct characteristics, including the unique genes of *trbA* and *ycjY* in pKF3-70, and the deletion of *traM* and *traS* in pKF3-140 (Figure 5B).

### The homology region between pKF3-94 and pC15-k

**Figure 6:**
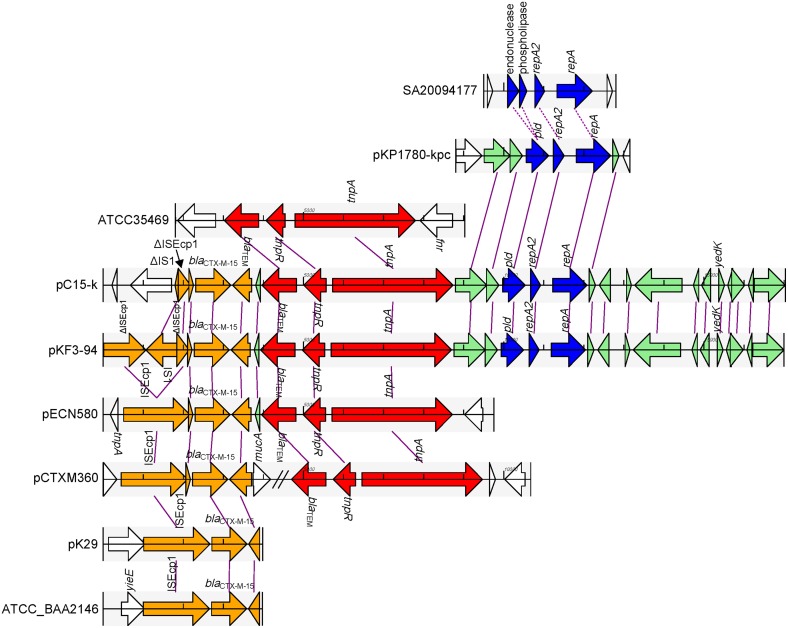
**Comparison of the 15 k homology region of pKF3-94 among the related plasmids**. The homologous genes are marked with the same color and connected together with lines, while non-homologous genes were left blank and unconnected. The regions from different origins are in different colors, with genes related to *bla*_CTX−M−15_ in orange, those related to *bla*_TEM_ in red, the replicon-related genes in blue, and other homologous genes in aqua.

pKF3-94 and pC15-k share a 15 kb homology region with a sequence identity of 99%. This region could be divided into two parts. One part contains resistance genes, including *bla*_CTX−M−15_ and *bla*_TEM_, and the other contains the FII_K2_ replicon (Figure 6). In addition, this latter part also contains a few MGEs that help to trace the formation of this homology region.

In the part containing resistance genes, *bla*_CTX−M−15_ is flanked by two truncated ISEcp1s (Figure 6). It has been demonstrated that ISEcp1 was able to mobilize an adjacent gene (*bla*_CTX−M−15_) (Zong et al., 2010; Dhanji et al., 2011). However, sequence analysis of this region in pKF3-94 showed that there is a complete IS1 (768 bp) inserted into ISEcp1 upstream of *bla*_CTX−M−15_, thereby dividing the complete ISEcp1 into two portions of 1057 and 309 bp, respectively. Further analysis showed that a sequence of 290 bp is deleted in the middle of ISEcp1, thereby rendering it non-functional. Interestingly, the ISEcp1 in pC15-k is shorter than that in pKF3-94 and only contains the truncated IS1 (80 bp, identical to the downstream side of IS1 in pKF3-94) and the truncated ISEcpl (309 bp, identical to downstream part of ISEcp1 in pKF3-94, Figure 6). This indicated that this region in pC15-k has been formed later than that in pKF3-94 and has probably arisen from a pKF3-94-like plasmid. Comparative genomicss showed that the complete sequence of ISEcp1 and *bla*_CTX−M−15_ are present in a few plasmid and bacterial genomes, such as in the plasmids pCTXM360 (from *K. pneumoniae*), pCTX-M3 (from *Citrobacter freundii*), pECL0701 (from *Enterobacter cloacae*), and pECN580 (from *E.coli*), and the chromosomes of *E.coli* JJ1886 and *K. pneumoniae* strains of ATCC BAA-2146, Kp13, and JM45. This finding suggests that the ISEcp1 enabled the spread of *bla*_CTX−M−15_ among a variety of plasmids and chromosomes in bacteria.

The other resistance gene, *bla*_TEM_, along with its adjacent genes of *tnpR* and *tnpA*, is associated with a Tn3 transposon (Figure 6). The *bla*_TEM_ gene encoding TEM β-lactamase and conferring resistance to the penicillin family of antibiotics was found in a group of three closely related transposons, Tn1, Tn2, and Tn3 (Bailey et al., 2011). This fragment of pKF3-94 (about 4.8 kb in length) is almost identical with that of pC15-k. The Tn3 transposon is incomplete, as its left side is shortened by 124 bp compared with the complete Tn3 found in the database. The deletion probably happened during recombination of Tn3 transposition. Complete or incomplete Tn3 transposons are present in a large number of plasmids such as pECN580 (incomplete, from *E. coli*), pKo6 (incomplete, from *K. pneumoniae*), pKOX_R1 (incomplete, from *K. oxytoca*), pCTXM360 (complete, from *K. pneumoniae*), and pCFSAN007428_01 (complete, from *Salmonella enterica*). A complete Tn3 transposon (4948 bp) was also found in the chromosomes of *Escherichia fergusonii* ATCC35469 and *Enterobacteria* phage P7, and showed nucleotide sequence identity of 100% compared to the corresponding region in pKF3-94. This suggests that the Tn3 transposon of pKF3-94 and pC15-k probably shares a common origin with those in the genomes of *E. fergusonii*, and *Enterobacteria* phage P7.

The replicon encoding part of the above mentioned 15 kb homology region is about 2.6 kb in length. It mainly encodes a FII_K2_ replicon containing *repA* (encoding RepA), *repA2* (encoding RepA2), and *pld* (Figure 6). An FII_K2_ replicon sequence has also been identified in *K. pneumoniae* plasmids such as pKP1780-kpc or pKpQIL-IT (FII_K2_ replicon, see Table 1; both with a nucleotide sequence identity of 99% compared to that of pKF3-94). No such FII_K2_ replicon is identified in *K. pneumoniae* chromosomes at present. Interestingly, a similar region was found in *Salmonella enterica* chromosomes such as SA20094177 (FII_S1_ replicon, see Table 1; with nucleotide sequence identity of 72%). Furthermore, the RepA and RepA2 proteins on pKF3-94 and SA20094177 show an amino acid sequence similarity of 91 and 75%, respectively. Sequence comparison shows that the phospholipase and endonuclease genes located upstream of *repA2* in SA20094177 are also similar to *pld* in pKF3-94 (with amino acid sequence similarity of 78 and 92%, respectively; Supplementary Figure S3). We deduce from this that the FII_*S*1_ replicon in SA20094177 and the FII_K2_ replicon in pKF3-94 probably evolved from a common origin.

Besides its presence in pKF3-94 and pC15-k, the part containing resistance genes was also found in some other plasmids, including pECN580, pKo6, and pKOX_R1. As shown in pKF3-94 and pC15-k, the two resistance genes (*bla*_CTX−M−15_ and *bla*_TEM_) and their related MGEs are situated next to each other while in the plasmid pCTXM360, they are separated by other sequences (Figure 6). The plasmid pKP1780-kpc only harbors the 2.6 kb part containing the replicon, suggesting that the two parts of the 15 kb region in pKF3-94 were probably derived from three different origins.

## Conclusion

Using the program Plasmid RST, we identified the replicons of the three IncF plasmids pKF3-70, pKF3-94, and pKF3-140. pKF3-70 is a one-replicon plasmid while pKF3-94 and pKF3-140 are both multi-replicon plasmids. Despite the fact that the three plasmids carry similar FII replicons of the same incompatibility group, the presence of the replicons of the other incompatibility groups in pKF3-94 and pKF3-140 enable them to co-exist in the same host and hence participate in widespread horizontal gene transfer.

Through extensive comparative genomics analysis, our work demonstrates that pKF3-94, pK1HV, and pC15-k share a common backbone structure but also contain their own variable regions. It is conceivable that the backbone structure of pC15-k and pK1HV is derived from that of a pKF3-94-like plasmid, and the variable regions are derived from a variety of plasmid or chromosomal sequences by HGT. The results lend further support for the role horizontal gene transfer plays in plasmid genome evolution and in the dissemination of genetic elements including drug resistance genes.

## Materials and methods

The host strain *K. pneumoniae* KF3 which harbored plasmid pKF3-94 was isolated from the laboratory of the First Affiliated Hospital of Wenzhou Medical University, Wenzhou, China in 2006. The plasmid pKF3-94 is one of three plasmids isolated from *K. pneumoniae* KF3. The other two plasmids named pKF3-70 (Yi et al., 2010) and pKF3-140 (Bai et al., 2013) have been reported earlier. Sanger sequencing was used to sequence three pKF3 plasmids. pKF3-94 was extracted using alkaline lysis method (Feliciello and Chinali, 1993). The purified plasmid DNA was sheared by a HydroShear DNA shearing device (volume, 200 ml; cycle number, 20; speed code, 7–8). Fragments of 1.6–3.0 Kb were recovered from agarose gel electrophoresis and ligated into a pUC18 vector. Clones were sequenced using an ABI 3730 automated sequencer. The derived sequences were assembled using the Phred/Phrap/Consed software package (http://www.phrap.org/phredphrapconsed.html).

The plasmids and chromosome genome sequences used in this study for comparative analysis and phylogenetic analysis were downloaded from the NCBI Database (http://www.ncbi.nlm.nih.gov). The 56 plasmids were selected based on the whole genome sequence (pKF3-94) comparison against the whole plasmids available in NCBI database with a cut-off value (max score) of about 8700. The accession numbers of the related plasmids and the chromosome sequences mainly mentioned in this paper were pKF3-94 (NC_013950.1/FJ876826.1), pKF3-70 (NC_013542.1), pKF3-140 (NC_013951.1), pK1HV (NC_020087.1/HF545434.1), pC15-k (NC_015154.1/HQ202266.1), pECN580 (KF914891.1), pCTXM360 (NC_011641.1), pK29 (EF382672.1), pKP1780-kpc (KF874497.2), CCBH10892 plasmid (KF727591.2), *E. fergusonii* ATCC35469 chromosome (CU928158.2), *K. pneumoniae* ATCC BAA-2146 (CP006659.1), and *S. enterica* subsp. SA20094177 (CP007468.1).

Multiple sequence alignments were performed using MAFFT (Katoh and Standley, 2013). Gene concatenation tree was reconstructed by Bayesian method using TOPALi v2 (Milne et al., 2009) and MrBayes (http://mrbayes.csit.fsu.edu/), incorporating the GTR+G model of nucleotide substitution. The generation number and sample frequency sampling were fixed to 2,000,000 and 100, respectively. Every clade was supported by Bayesian posterior probabilities (BPP). The best-fitting models [GTR+G model in Figure 2, HKY model in Figure 4B (R1) and Supplementary Figure S2 (R2, R3), K80 model in Figure 4B (R4)] of the nucleotide substitutions was selected using modelgenerator (Keane et al., 2006). Visualization and annotation of phylogenetic trees were done using Figtree (http://tree.bio.ed.ac.uk/software/figtree/). Potential open reading frames (ORFs) were predicted and annotated using Glimmer3 (http://www.cbcb.umd.edu/software/glimmer) and BASys (Van Domselaar et al., 2005), respectively. The Plasmid RST was identified with default parameters by Plasmid MLST Database (http://pubmlst.org/plasmid/; Updated in June, 2014). Comparisons of the nucleotide sequences were made using BlastN. Insertion sequences were predicted using ISFinder (Siguier et al., 2006). Orthologous groups of genes from plasmids (pKF3-94, pK1HV, pC15-k, etc.) were identified using BLASTp and inparanoid (Remm et al., 2001). Other bioinformatics software was written using Python (https://www.python.org/) and Biopython (Cock et al., 2009).

## Author contributions

JY designed and performed experiments, contributed to the interpretation of results, wrote the manuscript and approved the final version for publication; QB designed experiments, wrote the manuscript and approved the final version for publication. SW, KZ, ZW, WZ, YZ, MZ, CC, HW, HT, CZ, PL, JY, TX, HY, JL, LN, ZX, and JL approved the final version for publication.

### Conflict of interest statement

The authors declare that the research was conducted in the absence of any commercial or financial relationships that could be construed as a potential conflict of interest.

## Supplementary material

Supplementary Figure S1 **Collinearity analysis of the plasmid genomes of pKF3-94, pK1HV, pC15-k, pKF3-70, and pKF3-140**. The four different colors of lines indicate the orthologous genes between each plasmid pair.

Supplementary Figure S2 **The phylogenetic trees of R2 and R3**.

Supplementary Figure S3 **Amino acid sequences alignment of ***pld*** gene of pKF3-94 and phospholipase/endonuclease genes of SA20094177**. The identical amino acids are outlined with gray shadow, while the similar ones are outlined and left blank.
