# Supplementary figures and images for: Comparative genomics analysis of pKF3-94 in Klebsiella pneumoniae reveals plasmid compatibility and horizontal gene transfer
Source: Front Microbiol. 2015 Aug 18;6:831. doi: 10.3389/fmicb.2015.00831 (PMC4539522; doi:10.3389/fmicb.2015.00831)

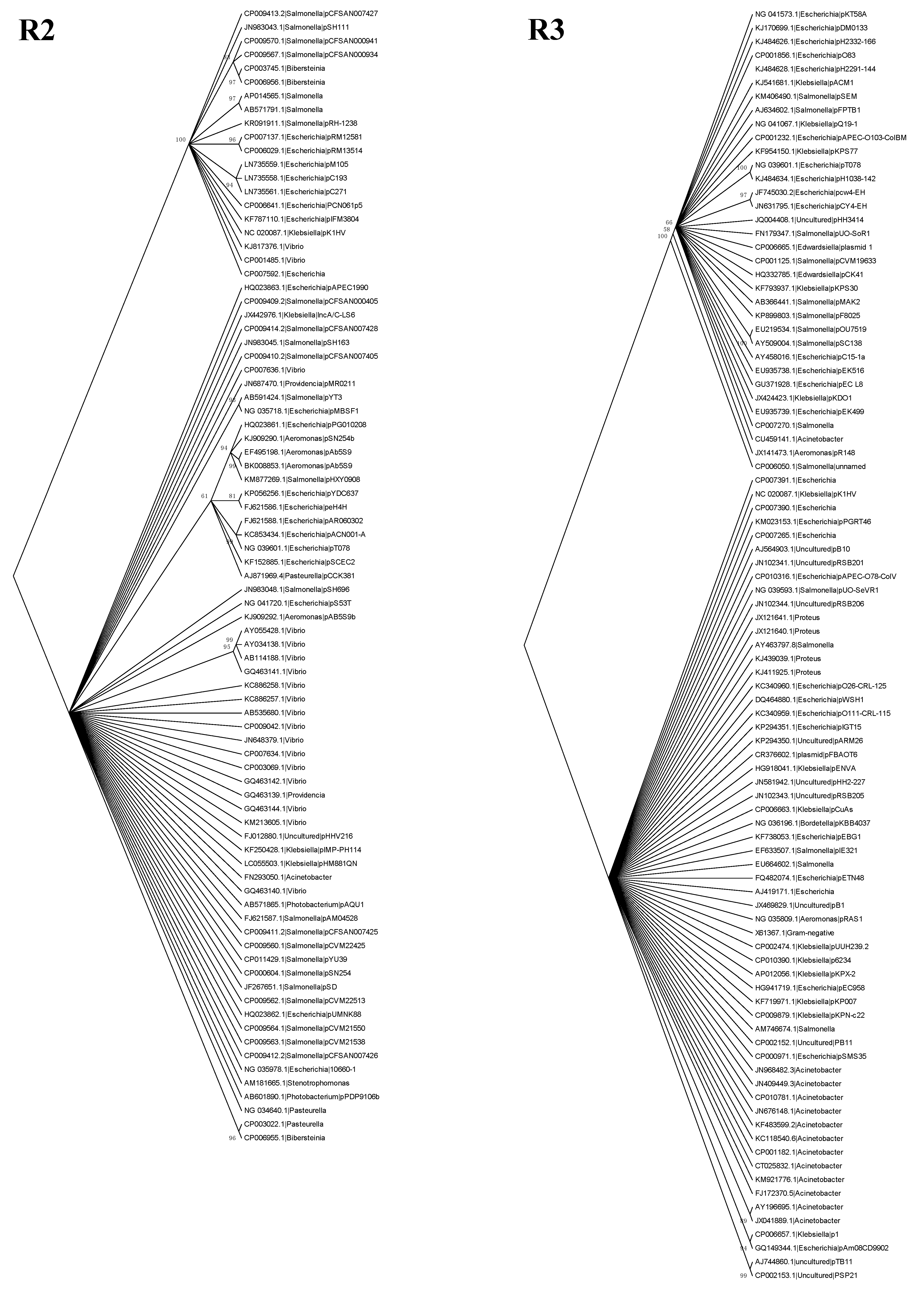

Supplement: Supplementary Figure S2 — The phylogenetic trees of R2 and R3. [file Image2.TIF]

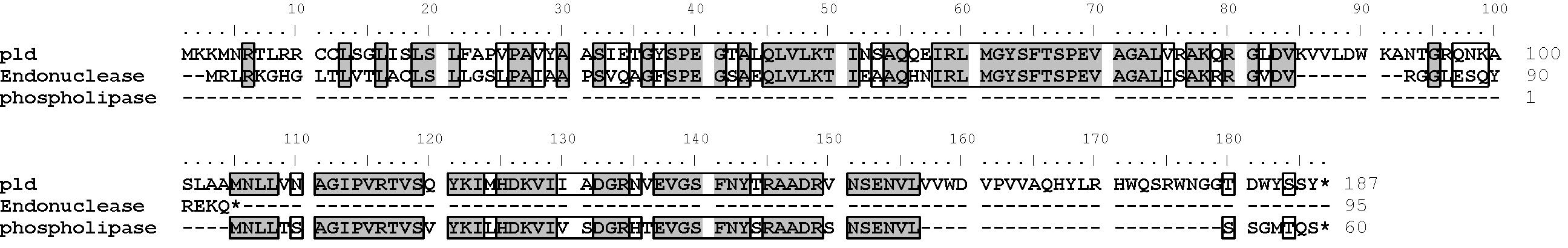

Supplement: Supplementary Figure S3 — Amino acid sequences alignment of pld gene of pKF3-94 and phospholipase/endonuclease genes of SA20094177. The identical amino acids are outlined with gray shadow, while the similar ones are outlined and left blank. [file Image3.TIFF]
